# Cognitive decline and alcohol consumption in the aging population—A longitudinal analysis of the Survey of Health, Ageing and Retirement in Europe

**DOI:** 10.1192/j.eurpsy.2022.2344

**Published:** 2022-11-18

**Authors:** Stephan Listabarth, Magdalena Groemer, Thomas Waldhoer, Benjamin Vyssoki, Nathalie Pruckner, Sandra Vyssoki, Alexander Glahn, Deirdre Maria König-Castillo, Daniel König

**Affiliations:** 1 Clinical Division of Social Psychiatry, Department of Psychiatry and Psychotherapy, Medical University of Vienna, Vienna, Austria; 2 Department of Epidemiology, Center for Public Health, Medical University of Vienna, Vienna, Austria; 3 Department of Health Sciences, St. Pölten University of Applied Sciences, Sankt Pölten, Austria; 4 Department for Psychiatry, Social Psychiatry and Psychotherapy, Medical University of Hannover, Hannover, Germany; 5 Department of Urology, Medical University of Vienna, Vienna, Austria

**Keywords:** Aged, alcohol use disorder, dementia, epidemiology, mild cognitive impairment

## Abstract

**Background:**

Prevalence of cognitive decline and dementia is rising globally, with more than 10 million new cases every year. These conditions cause a significant burden for individuals, their caregivers, and health care systems. As no causal treatment for dementia exists, prevention of cognitive decline is of utmost importance. Notably, alcohol is among the most significant modifiable risk factors for cognitive decline.

**Methods:**

Longitudinal data across 15 years on 6,967 individuals of the Survey of Health, Ageing and Retirement in Europe were used to analyze the effect of alcohol consumption and further modifiable (i.e., smoking, depression, and educational obtainment) and non-modifiable risk factors (sex and age) on cognitive functioning (i.e., memory and verbal fluency). For this, a generalized estimating equation linear model was estimated for every cognitive test domain assessed.

**Results:**

Consistent results were revealed in all three regression models: A nonlinear association between alcohol consumption and cognitive decline was found—moderate alcohol intake was associated with overall better global cognitive function than low or elevated alcohol consumption or complete abstinence. Furthermore, female sex and higher educational obtainment were associated with better cognitive function, whereas higher age and depression were associated with a decline in cognitive functioning. No significant association was found for smoking.

**Conclusion:**

Our data indicate that alcohol use is a relevant risk factor for cognitive decline in older adults. Furthermore, evidence-based therapeutic concepts to reduce alcohol consumption exist and should be of primary interest in prevention measures considering the aging European population.

## Introduction

Prevalence rates of cognitive decline and dementia are rising globally—concurrent with the proportion of the population of ≥50-year-olds (older adults) and ≥65-year-olds (senior adults). The worldwide incidence of cognitive decline and dementia is estimated at nearly 10 million new cases every year [1]. Importantly, cognitive decline causes a significant burden on the affected individuals and their families and caregivers. Additionally, dementia represents a significant social and economic burden due to high medical and informal care costs. In 2015, the estimated global societal cost of dementia was equivalent to 1.1% of the global gross domestic product (GDP) [1].

Prevention is of utmost importance as no causal therapy exists for dementia and cognitive decline. The *Lancet Commission* recently listed modifiable risk factors for dementia in its “*Dementia prevention, intervention, and care 2020 report*,” including excessive alcohol consumption, low educational obtainment, depression, and smoking. Modifying said risk factors is suggested to prevent or delay up to 40% of the cases of cognitive decline (as in dementia) [2].

It is a well-established fact that excessive consumption of alcohol (≥3 drinks/day) over an extended period can lead to irreversible structural damage to the brain and, subsequently, to a decline in cognitive and executive functions [3, 4]. Long-term increased alcohol consumption is known to cause extensive white and gray matter loss, severe vitamin B1 (thiamine) deficiency, and, consequently, an increased risk for vascular dementia, Alzheimer’s disease (AD) and Wernicke–Korsakoff encephalopathy [5–9]. However, the prevalence of health-detrimental alcohol consumption has been shown to increase in the general population and, importantly, in the older and senior adults especially [10–13]. As senior adults are postulated to be more prone to alcohol-associated brain damage due to their potential pre-existing diseases and, in further consequence, reduced alcohol metabolism [14], this may further compound the risk of cognitive decline in the population already most affected by cognitive decline.

Conversely, numerous studies suggest a possible link between low-to-moderate alcohol consumption (≤2 drinks/day for men and ≤1 drink/day for women) and enhanced cognitive function among older adults compared to non-alcohol consumers [4, 8, 15–24]. These studies propose a “U-shaped” association between alcohol drinking and cognitive decline, similar to that seen in cardiovascular disease [25, 26]. Whereas moderate alcohol intake may be associated with overall better global cognitive function, complete abstinence from alcohol is known to correlate with poorer cognitive outcomes over the course of life [24, 27, 28].

Considering the limited therapeutic options, the high socioeconomic impact, as well as the drastic consequences cognitive decline and dementia have on affected individuals, it is of high clinical relevance to gain clear evidence in the controversial discussion on the influence of alcohol on cognitive functioning. Therefore, this study aimed to examine the possible effect of alcohol consumption on cognitive decline in the aging population by analyzing one of the most comprehensive longitudinal transnational datasets on an individual level. Furthermore, the effect of the modifiable risk factor alcohol consumption (for which evidence-based therapeutic concepts exist) and other modifiable (smoking, depression, and educational obtainment) as well as non-modifiable risk factors (age and sex) were compared.

## Methods

### Data

#### Study population

The study population consists of participants of the “Survey of Health, Ageing and Retirement in Europe” (SHARE, wave 1–7, release 7.1.0) [29]. The SHARE database provides data on health, socioeconomic status, and social/family networks of individuals of 50 years of age and above in 27 European countries and Israel. The first wave of the SHARE survey was conducted in 2004. Thereafter, further waves followed periodically, with the last wave (wave 7) included in the present analysis, which took place from 2017 to 2019. Collectively, this amounts to longitudinal data on cognitive function and potential risk factors over the period of 15 years. All participants completing wave 1 to wave 7 and all relevant data available were included in the present analysis.

#### Assessments

Data collection was undertaken by computer-assisted personal interviewing, that is, face-to-face interviews with digital support (see [30] for methodological details). As primary variables operationalizing *cognitive function*, the results of the 10-word recall test and the verbal fluency test, both often used as indicators for the presence of cognitive impairment and dementia in previous studies, were chosen. For the first test, participants were asked to repeat a list of 10 words (a) immediately after the acoustical presentation and (b) after a time delay. The total score of the recall test for both timepoints ranges from 0 to 10, with one point for each correctly enlisted word. For the latter test of verbal fluency—an assessment of executive functioning—participants were asked to enlist as many terms as possible for a specific semantic category (e.g., “animals”). Accordingly, the total score of the verbal fluency test is the sum of any correct word stated by the participant.

To assess *alcohol consumption,* participants were asked for the average number of days a week with alcohol consumption, yielding the following seven categories: “almost every day,” “5 or 6 days a week,” “3 or 4 days a week,” “once or twice a week,” “one or twice a month,” “less than once a month,” and “not at all in the last 6 months.” Observations with “refusal” or “do not know” were set to missing and excluded from the analysis. Additionally, participants were asked whether excessive drinking has ever been a problem throughout their lives. The level of *educational attainment* was operationalized by the total number of years of full-time education received by the participants. Furthermore, to assess the risk factor of *smoking,* participants were asked to confirm whether they have ever smoked cigarettes, cigars, cigarillos, or a pipe daily for at least 1 year. As generally known, depression can also interfere with cognitive functioning. Thus, the Euro-Depression-Scale-Score (EURO-D) [31] was included in the analysis as an indicator of depressive symptoms. This scale is comprised of 16 items evaluating a variety of depressive symptoms and eventually yields a total score ranging from a minimum of 0 points (“not depressed at all”) and a maximum score of 12 points (“very depressed”). Further details regarding operationalization and actual assessment of the primary and secondary variables used in this analysis can be found in the manual “Scales and Multi-Item-Indicators” of the SHARE project [32].

### Statistical analysis

The effect of the variables wave (1–7 [corresponding to the variable time]), sex, age at first wave (years), education (years), depression score (0–6), alcohol consumption at baseline (wave 1), smoking (yes/no) at baseline, and country on the three outcome variables (i.e., verbal fluency, short-term verbal memory, and delayed verbal memory) was modeled by employing a generalized estimating equations (GEE) model using a linear link function in SAS version 9.4 (SAS Institute Inc., Cary, NC). All observations per participant were included except those where there was no response at that wave.

All variables were treated as categorical except the continuous variables education and age, which are given in years. Reference groups were set as follows: wave (1), sex (males), smoking (no), depression (0), alcohol (7 = not at all in the last 6 months), and country (1 = Austria). For the analysis, the effect of depression is presented in six categories, each comprised of two of the 12 levels of the EURO-D scale. The estimated effect of age is presented for a 10-year difference in the figures, since the effect in absolute terms is small. Results are shown along with 95% confidence intervals. Three models (verbal fluency, verbal memory, and delayed verbal memory) were estimated. The significance level for Type 3 GEE statistics was set using Bonferroni rule to 5%/3 = 0.0167 to acknowledge multiple testing. Though *p*-values are adjusted for multiple testing, the analysis is exploratory in nature and the observed associations should not be interpreted in a causal manner.

## Results

### Descriptive analysis

A total of 9,461 individuals participated in all seven waves. For 6,967 of those individuals, data were available for the risk factors of interest and were, thus, included in the final analysis. Characteristics of the study population at baseline (i.e., wave 1) are described in Table 1. In total, 3,373 women (48.4% of the total study population) and 3,593 men were included in the present study, with a mean age of 60 years. Daily alcohol consumption was reported by 28.9% of the study population and an additional 40.9% reported alcohol consumption at least once or twice a week. Conversely, 18.6% of the study population reported drinking alcohol only two times or more seldom per month, whereas 11.7% stated complete abstinence. Moreover, 1.95% of the study population reported having had a history of excessive drinking in the past. In addition, 51.5% of the participants had been smoking cigarettes (or other tobacco products) daily for at least 1 year of their life. The average length of full-time education (including school) was 10.8 years. The study population comprised individuals from 10 European countries and Israel, with the highest share of participants coming from Belgium (17.5%), Greece (14.4%), and Sweden (13.5%). The evaluation of depressive symptoms using the EURO-D scale yielded a fraction of 30.8% of the study population reaching a scale score higher than four, indicating a state of mild to severe depression.

**Table 1.** Characteristics of study population at baseline visit.

Characteristic	Participants (*n* = 6,967)
Sex	*n* (%)
Male	3,594 (51.59)
Female	3,373 (48.41)
	Mean (standard deviation)
Age	59.95 (7.93)
Years of education	10.87 (4.27)
Euro-Depression-Score	1.86 (1.78)
Smoking status	*n* (%)
Non-smoking	3,377 (48.47)
Smoking	3,583 (51.43)
Missing	7 (0.10)
Alcohol consumption	
“Almost every day”	2,012 (28.91)
“5 or 6 days a week”	214 (3.07)
“3 or 4 days a week”	766 (11.01)
“Once or twice a week”	1,864 (26.78)
“Once or twice a month”	858 (12.33)
“Less than once a month”	434 (6.24)
“Not at all in the last 6 months”	812 (11.67)
Lifetime history of excessive drinking (obtained at wave 4)	
Yes	136 (1.95)
No	4,504 (64.65)
Missing	2,327 (33.4)
Country	
Austria	380 (5.45)
Germany	456 (6.55)
Sweden	943 (13.54)
Spain	407 (5.84)
Italy	638 (9.16)
France	784 (11.25)
Denmark	668 (9.59)
Greece	1,000 (14.35)
Switzerland	323 (4.64)
Belgium	1,220 (17.51)
Israel	148 (2.12)

### Statistical analysis

**Figure 1.**
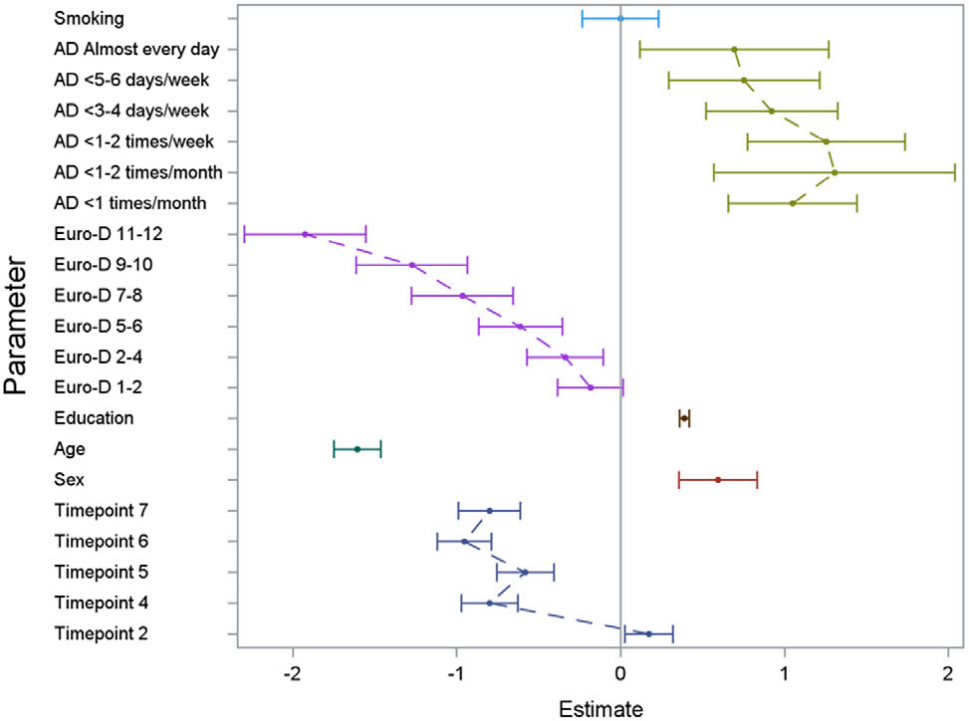
Results of the GEE model illustrating the effect of the analyzed risk factors on the cognitive dimension verbal fluency. For the set reference levels, see the section “Statistical analysis.” AD, consumption of alcoholic drinks; Euro-D, Euro-Depression-Scale-Score; education, years of education; age, age at baseline in groups of 10 years; timepoint, number of follow-up interview in reference to the baseline interview.

**Figure 2.**
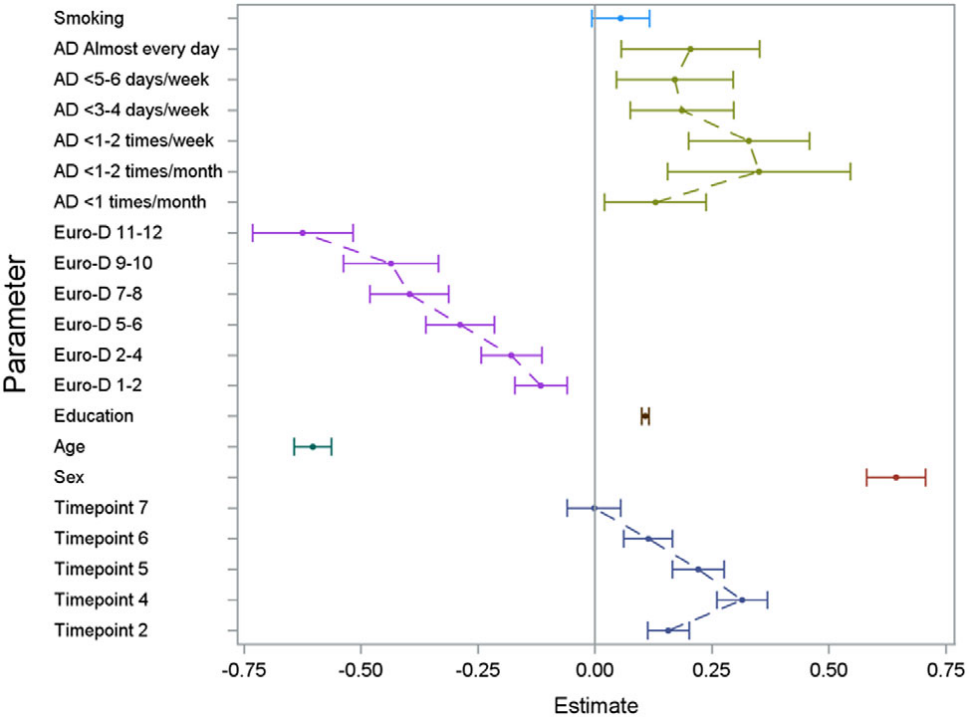
Results of the GEE model illustrating the effect of the analyzed risk factors on the cognitive dimension verbal memory (short term). For the set reference levels, see the section “Statistical analysis.” AD, consumption of alcoholic drinks; Euro-D, Euro-Depression-Scale-Score; education, years of education; age, age at baseline in groups of 10 years; timepoint, number of follow-up interview in reference to the baseline interview.

**Figure 3.**
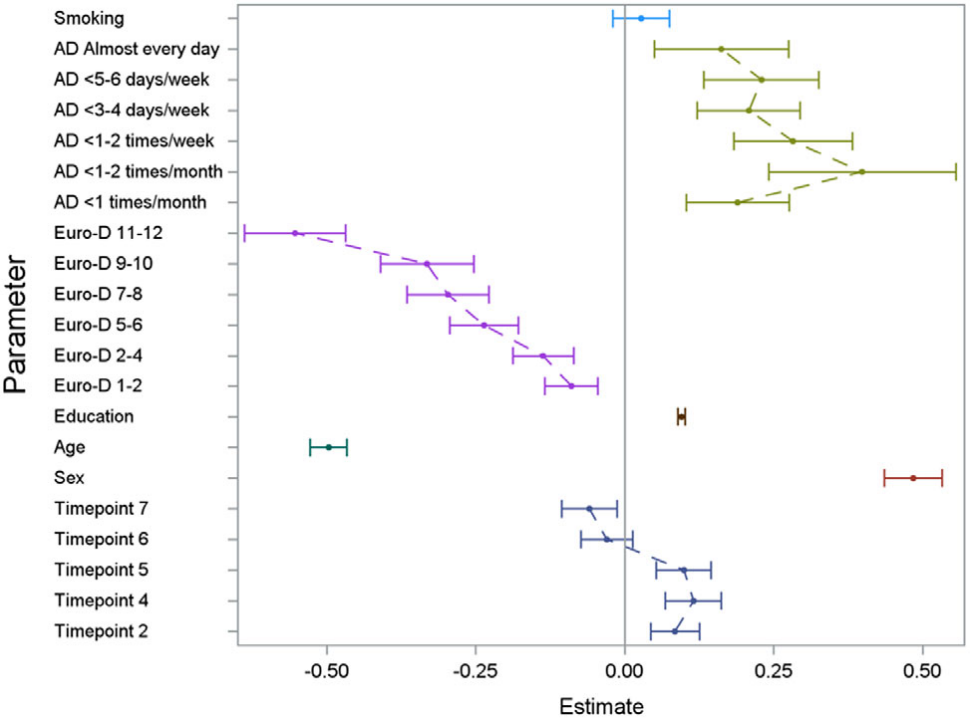
Results of the GEE model illustrating the effect of the analyzed risk factors on the cognitive dimension of delayed verbal memory. For the set reference levels, see the section “Statistical analysis.” AD, consumption of alcoholic drinks; Euro-D, Euro-Depression-Scale-Score; education, years of education; age, age at baseline in groups of 10 years; timepoint, number of follow-up interview in reference to the baseline interview.

The regression model on the three outcome variables (verbal fluency, verbal memory, and delayed verbal memory score) yielded consistent results (see Figures 1– 3 and Supplementary Tables 1 and 2) with significant effects of alcohol consumption, sex, age, educational level, depressive symptoms, country, and time since first wave. In Figures 1– 3 (showing the results for the three different cognitive assessments), the effect of the single categorical risk factors is depicted in comparison to the corresponding reference groups (see the section “Statistical analysis”). Thus, a nonlinear effect—similar to the hypothesized U-shaped association—of alcohol consumption on cognitive function was revealed, with peak cognitive functioning in individuals exhibiting moderate alcohol consumption patterns (i.e., 1 or 2 days a month consuming alcoholic drinks). Individuals sustaining complete alcohol abstinence scored worst in cognitive function. However, with an increased frequency of alcohol consumption (consuming alcoholic drinks three to four times per week or more), the observed positive effect on cognitive function decreased compared to moderate drinking patterns. Furthermore, positive effects on cognitive function were associated with a higher educational level and female sex, while older age and an increase in depressive symptoms affected cognitive function negatively. No significant effect was found for smoking status.

An extended analysis, considering the history of lifetime excessive drinking as the primary indicator for alcohol consumption, yielded almost identical results (see Supplementary Figures S1–S3), thus indicating that the observed effects on cognitive functioning can be interpreted as function of both, frequency and quantity of alcohol consumption.

We repeated the analysis for individuals who participated in the first two waves only to check whether early withdrawals may bias the analyses. However, the results mirrored the analyses of those participants with data including up to seven waves and therefore are not presented.

## Discussion

The aim of the present analysis was to examine the effect of alcohol consumption and further modifiable risk factors on cognitive decline in individuals aged 50 years or older in a longitudinal, multi-national sample.

The most interesting finding of this study was the revealed nonlinear effect of alcohol consumption on cognitive function: The best cognitive performance was found in individuals with low-to-moderate alcohol consumption, whereas less favorable values were found in those individuals with (almost) daily alcohol consumption. Notably, the least favorable cognitive performance was revealed in individuals who reported complete alcohol abstinence. While this finding may at first seem counterintuitive—considering the well-known detrimental effects of alcohol on the central nervous system and cognitive function—it agrees with previous literature. The number of studies that support the claim that *moderate* alcohol drinking is beneficial to cognitive health outweighs the number of studies that found a decline in cognitive function [4, 22, 24]. The following explanations for this observed association are suggested:

First, there has been the hypothesis of less harmful or even beneficial effects of alcoholic beverages (due to specific compounds and ingredients, such as flavonoids) on the central nervous system. Thus, some studies found moderate wine consumption, specifically red wine, to be the most favorable regarding cognitive amelioration and prevention of dementia [33, 34]. However, contradictory studies challenge this theory, finding no association between low-to-moderate alcohol consumption and protection against neurocognitive decline [35]. Furthermore, other studies have shown that even moderate alcohol intake promotes adverse changes to the brain, including hippocampal atrophy [9, 36, 37].

Second, it has been postulated that the observed nonlinear association may be due to the so-called “abstainer bias,” meaning that abstainers within the study cohort may be more severely ill and thus, do not (or are not able to) engage in alcohol consumption [35, 38]. Especially within the study sample of our present study, this latter explanation may be probable, considering the advanced average age of about 60 years at baseline assessment. Importantly, this assertion agrees with the further analyses performed: When the history of lifetime extensive drinking had been evaluated for a possible effect on cognitive function, congruent results with the primary analyses were revealed.

In addition to the effect of alcohol, also effects of educational level, age, and depressive symptoms on cognitive function were revealed in the present study. A higher level of educational obtainment was positively associated with cognitive functioning. This finding resonates well with previous research, in which the protective effect of higher-level education was reported [39, 40]. Previous explanations for this association between reduced risk for cognitive decline or dementia and a higher degree of educational obtainment hypothesized that this cognitive exertion in early- and mid-life stages might result in an increased cognitive reserve in older age. A further explanation may be that individuals with higher levels of educational obtainment may engage in mentally stimulatory activities more frequently, especially in later life stages, contributing to the preservation of cognitive function and, thus, impeding the onset of cognitive decline [41–43].

The observed association between cognitive decline and increasing age within our cohort corresponds to findings from the literature, showing a heightened prevalence of cognitive decline and dementia in older adults [44]. Notably, the amount of late-life depressive symptoms was nearly linearly associated with cognitive function and turned out to be one of the most substantial factors in the present study. This finding is in line with previous literature, as reflected by an extensive meta-analysis of 23 cohort studies that reported this effect as consistent [45]. However, the relationship between depression and cognitive functioning might also be bidirectional and was therefore not interpreted as causal. While depressive symptoms are reported to represent a risk factor for cognitive decline—a condition that historically was described as “pseudodementia” [46], impaired cognitive functioning may, in turn, also be leading to the emergence of depressive symptoms [47].

As there is no rational explanation for the observed effect of the variable “country” on cognitive functioning found within the present study population, this effect may either be interpreted as an indication of the presence of an, as yet, unknown country-specific influence. However, it may also be due to the significantly different number of recruited participants per country (see Table 1). Similarly, the effect of the variable time passed since the beginning of the observation period (i.e., Timepoint 2, Timepoint 3, and later) may be due to collinearity between time progression and the participants’ increasing age.

Interestingly, the effect of sex found in the present study, which revealed female individuals in the study to have reached significantly higher scores on verbal fluency and memory tests, contradicts previous literature. For example, in a recent review by Laws et al. [48], only one study [49] reported results in which females outperformed their male counterparts in categorical (verbal) fluency. However, different explanations for these sex differences are discussed. Whereas some literature highlights the role of sex hormones on cognitive functioning throughout different stages of life [50], other studies claim that sex differences occur primarily due to differences between particular cognitive domains (e.g., verbal versus visuospatial abilities) [51, 52]. Such sex dimorphisms also exist in other neurodegenerative disorders and are, in part, hypothesized to be influenced by immunomodulatory properties of sex hormones (principally 17β-estradiol, which promotes neuroinflammatory processes) [53, 54]. Similar to AD, a higher prevalence in females is also postulated for patients with multiple sclerosis (MS), whereas for Parkinson’s disease (PD), Lewy body dementia, and motor neuron diseases such as amyotrophic lateral sclerosis are more prevalent in males [55–57]. Regarding trajectories of cognitive decline, patients with neurodegenerative disorders typically show gradual deterioration of cognitive function over time and with progression of the disease [58]. However, knowledge on the specific time courses within different forms of neurodegeneration are still limited, and nonlinear and atypical progressions are postulated [59]: Whereas most patients with AD and PD exhibit a cognitive decline over the course of a decade or more, cognitive functioning may remain stable in patients with MS [60–62]. In contrast to the previously discussed diagnoses, vascular dementia, exhibits a more ambiguous pattern and occurs more frequently in males under and females over the age of 80, who then tend to have a less favorable outcome than their male counterparts [63, 64].

The following limitations of the present study need to be addressed: The quality of data based on surveys by questionnaires depends on honest and thorough replies by the participants—especially concerning stigmatized topics, such as alcohol consumption. This, in turn, may lead to reporting bias, potentially distorting the results. Second, even though hardly feasible, the ideal dataset for this kind of analysis would comprise data on risk factors and cognitive performance throughout an individuals’ whole lifespan. Nevertheless, the individual datasets used for the present study include evaluating potential risk factors as a part of a baseline assessment at an advanced age (average around 60 years) and an evaluation of cognitive functioning at baseline and at subsequent timepoints within 15 years. A further limitation due to feasibility constraints concerns the evaluation of cognitive functioning. A comprehensive neuropsychological test battery would represent the gold standard but is hardly attainable when aiming for a study sample of size large as that used for the present study. Furthermore, it is not feasible to consider all risk factors suggested for cognitive decline within one analysis. Thus, the present study focuses on the effect of alcohol consumption, also considering a selection of major sociodemographic and socioeconomic variables. A further important limitation of the present manuscript is the lack of information on the *quantity* of alcohol ingested. However, as the necessary data to quantify alcohol ingestion in drinks/grams per week had only been assessed at the sixth and seventh wave of the SHARE survey, the analyses were constrained in their applicability due to the short period which could be assessed (maximum of 2 years) and were therefore not included in the manuscript.

Notwithstanding these limitations, the present study provides coherent results regarding the effect of alcohol consumption on three cognitive dimensions based on a sizeable transnational study sample. Moreover, it takes additional essential risk factors into account, thus, allowing an assessment of the strength of the effects of the particular risk factors.

## Conclusion

The main goal of this study was to determine the effect of alcohol consumption in the aging population on cognitive functioning by analyzing longitudinal data of a large transnational study population and considering further known risk factors for cognitive decline and dementia. In line with previous literature, we found a nonlinear association with low-to-moderate alcohol consumption favorably affecting cognitive functioning, measured by verbal memory and fluency tests. However, compared to other modifiable (e.g., level of education and depressive symptoms) and non-modifiable risk factors (e.g., sex and age), the observed positive effects of low-to-moderate alcohol consumption are rather small. Considering this and a potential bias due to unmeasured confounders subsumed under the term “abstainer bias,” it seems more than questionable to claim that regular alcohol consumption—on any level—is protective in regard to cognitive decline in older adults; not to mention somatic complications, such as alcoholic liver diseases, which can be induced by chronic alcohol use. All in all, this study backs existing research by highlighting the complex interplay of multiple factors that contribute to cognitive decline and the development of dementia. Further focusing on the role of alcohol consumption in cognitive health and disease, future research should aim to identify the exact mechanisms by which alcohol potentially promotes or impairs cognitive functioning and, consequently, allow for more clear-cut evidence-based recommendations on alcohol use, especially in older adults.

## Data Availability

The data that support the findings of this study are available from SHARE-ERIC. Restrictions apply to the availability of these data, which were used under license for this study. Data are available at http://www.share-project.org with the permission of SHARE-ERIC.

## Supplementary material

For supplementary material accompanying this paper visit http://doi.org/10.1192/j.eurpsy.2022.2344. click here to view supplementary material
