# Supplementary material for: Cognitive decline and alcohol consumption in the aging population—A longitudinal analysis of the Survey of Health, Ageing and Retirement in Europe
Source: Eur Psychiatry. 2022 Nov 18;65(1):e83. doi: 10.1192/j.eurpsy.2022.2344 (PMC9748981; doi:10.1192/j.eurpsy.2022.2344)
Supplement: Supplementary file 1 [file epasup.zip › S0924933822023446sup002.docx]

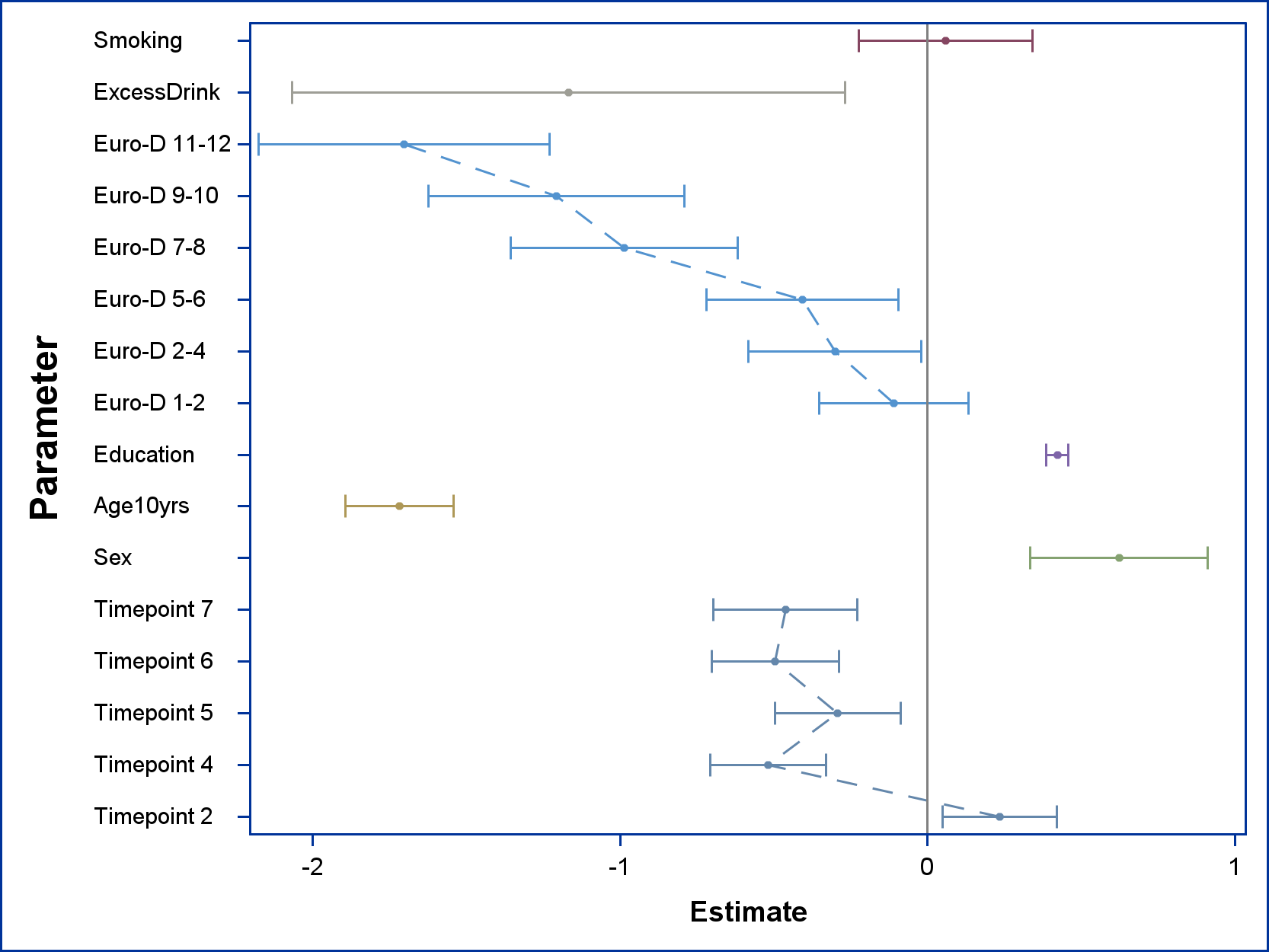


Abbildung Figure S1: Results of the GEE-Model illustrating the effect of the analyzed risk factors on the cognitive dimension verbal fluency. Smoking (Reference level = “No”), ExcessDrink (Lifetime history of excessive drinking, reference level = “No”), Euro-D (Euro-Depression-Scale-Score, reference level = 0), Education (Years of education, continuous variable), Age (Age at baseline in groups of 10 years, for statistical modelling treated as continuous variable), Timepoint (Number of follow-up-Interview, reference level = “Wave 1 / baseline interviews”)


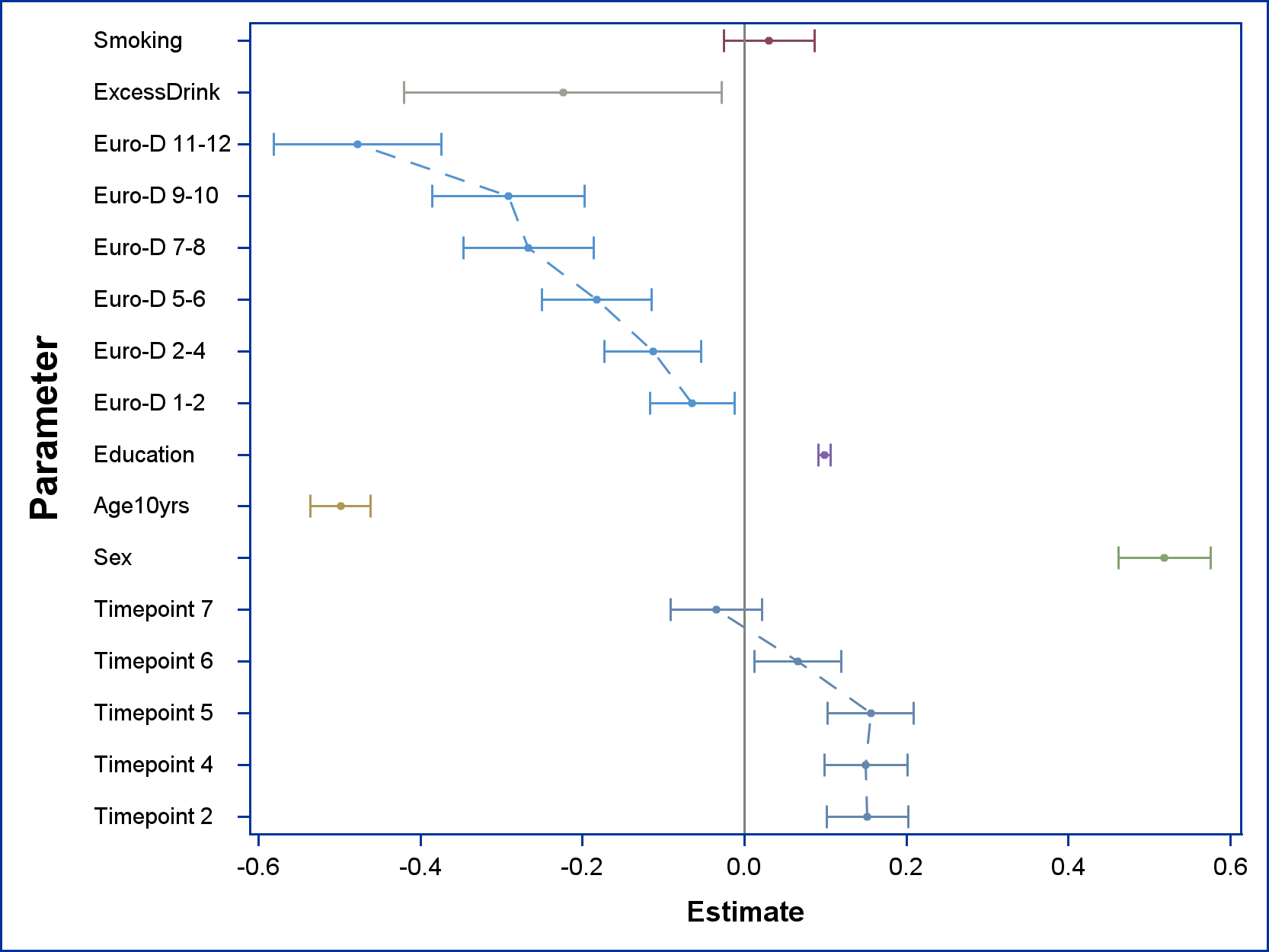


Abbildung Figure S2: Results of the GEE-Model illustrating the effect of the analyzed risk factors on the cognitive dimension verbal memory (short term). Smoking (Reference level = “No”), ExcessDrink (Lifetime history of excessive drinking, reference level = “No”), Euro-D (Euro-Depression-Scale-Score, reference level = 0), Education (Years of education, continuous variable), Age (Age at baseline in groups of 10 years, for statistical modelling treated as continuous variable), Timepoint (Number of follow-up-Interview, reference level = “Wave 1 / baseline interviews”)


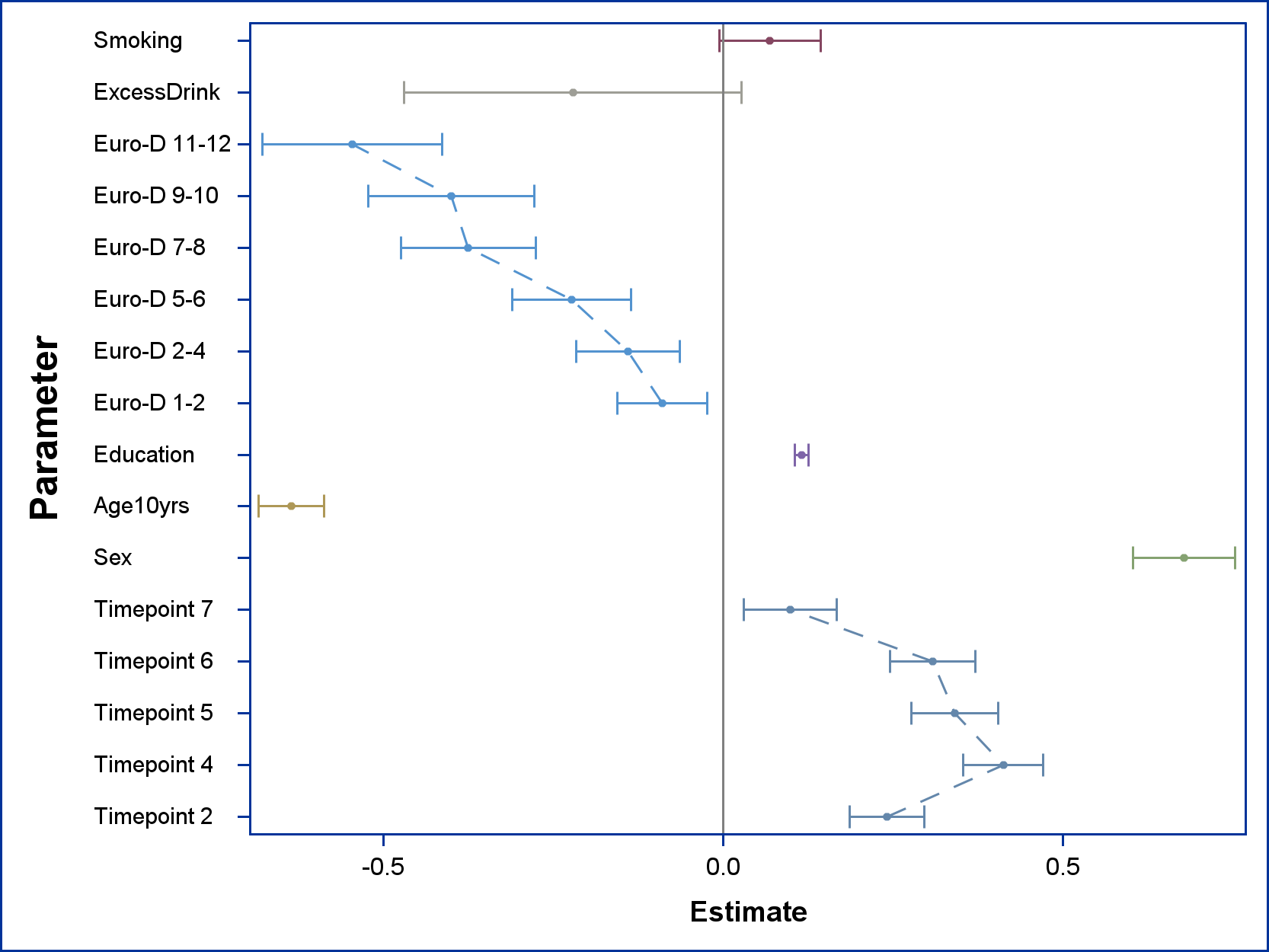


Abbildung Figure S3: Results of the GEE-Model illustrating the effect of the analyzed risk factors on the cognitive dimension delayed verbal memory. Smoking (Reference level = “No”), ExcessDrink (Lifetime history of excessive drinking, reference level = “No”), Euro-D (Euro-Depression-Scale-Score, reference level = 0), Education (Years of education, continuous variable), Age (Age at baseline in groups of 10 years, for statistical modelling treated as continuous variable), Timepoint (Number of follow-up-Interview, reference level = “Wave 1 / baseline interviews”)
